# Potential for Isolation of Immortalized Hepatocyte Cell Lines by Liver-Directed *In Vivo* Gene Delivery of Transposons in Mice

**DOI:** 10.1155/2019/5129526

**Published:** 2019-06-02

**Authors:** Masahiro Sato, Issei Saitoh, Emi Inada, Shingo Nakamura, Satoshi Watanabe

**Affiliations:** ^1^Section of Gene Expression Regulation, Frontier Science Research Center, Kagoshima University, Kagoshima 890-8544, Japan; ^2^Division of Pediatric Dentistry, Graduate School of Medical and Dental Science, Niigata University, Niigata 951-8514, Japan; ^3^Department of Pediatric Dentistry, Graduate School of Medical and Dental Sciences, Kagoshima University, Kagoshima 890-8544, Japan; ^4^Division of Biomedical Engineering, National Defense Medical College Research Institute, Saitama 359-8513, Japan; ^5^Animal Genome Unit, Institute of Livestock and Grassland Science, National Agriculture and Food Research Organization (NARO), Tsukuba, Ibaraki 305-0901, Japan

## Abstract

Isolation of hepatocytes and their culture *in vitro* represent important avenues to explore the function of such cells. However, these studies are often difficult to perform because of the inability of hepatocytes to proliferate *in vitro*. Immortalization of isolated hepatocytes is thus an important step toward continuous *in vitro* culture. For cellular immortalization, integration of relevant genes into the host chromosomes is a prerequisite. Transposons, which are mobile genetic elements, are known to facilitate integration of genes of interest (GOI) into chromosomes *in vitro* and *in vivo*. Here, we proposed that a combination of transposon- and liver-directed introduction of nucleic acids may confer acquisition of unlimited cellular proliferative potential on hepatocytes, enabling the possible isolation of immortalized hepatocyte cell lines, which has often failed using more traditional immortalization methods.

## 1. Introduction

It is well-known that primary cells can only undergo a limited number of cell divisions in culture, although the number varies according to species, cell type, and culture conditions [1]. Cells at a state where they can no longer divide are referred to as being in “replicative senescence,” which is characterized by changes in cellular morphology, as exemplified by enlarged cell size and formation of multiple nuclei [2, 3]. Replicative senescence is also associated with the activation of tumor suppressor genes, such as *p53*, retinoblastoma (*RB*), and *p16*. However, this replicative senescence can be overcome by overexpression of viral genes such as simian virus 40 large T antigen (SV40T) [4] or telomerase reverse transcriptase protein (TERT), the latter being responsible for elongation of telomeres [5, 6], through *in vitro* transfection of primary cells. It is known that expression of SV40T causes inactivation of p53 [7]. As a result, cells acquiring continuous cell division capacity can retain many of the original and relevant characteristics of the source tissue. These types of cells are generally called “immortalized cells.”

Immortalized cells can also be acquired from transgenic (Tg) mice that harbor a temperature-sensitive *SV40T* gene called tsA58 [8, 9]. Mutant SV40T is inactive at 39°C; therefore, the gene functions normally in Tg mice. However, at 33°C, which is below the normal physiological temperature, the gene becomes active. Therefore, when researchers dissect cells from the target organs/tissues of this Tg line for *in vitro* primary culture, these primary cells must always be cultured under low-temperature conditions to ensure that the temperature-sensitive *SV40T* gene is activated, allowing unlimited cell proliferation. According to Obinata's review [10], numerous different cell lines have been established using this Tg system, including bone marrow stromal cell lines (TBR series), a stromal cell-dependent hematopoietic stem-like cell line (THS-119), a dendritic cell line (SVDC), a hepatocyte cell line (TLR), a Leydig cell line (TTE1), and a Sertoli cell line (TTE3). One of the drawbacks associated with this system is the initial establishment of the Tg line, which requires considerable time and cost. Additionally, if they are subjected to in-house breeding, animal maintenance and genotyping are required, which are laborious and expensive. Similarly, fibroblasts derived from *p53* knockout (KO) mice proliferate continuously without showing aging or crisis. However, cardiac muscle cells or hepatocytes isolated from such *p53* KO mice fail to proliferate indefinitely [11].

**Figure 1:**
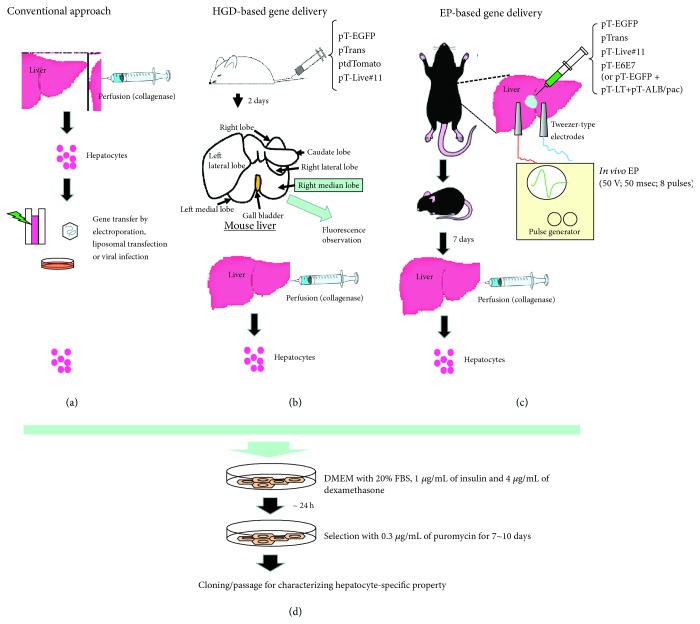
Methods for establishing hepatocyte cell lines using conventional approaches (a), HGD-based gene delivery (b) and EP-based gene delivery (c). In (a), the liver is first perfused with collagenase to isolate single hepatocytes, to which *in vitro* gene delivery using EP, liposomes, or virus is applied. In (b), HGD is performed with transposon vectors, and 2 days later, perfusion with collagenase is performed to isolate single hepatocytes. In (c), transposon vectors are directly introduced into the parenchyma of the livers of anesthetized mice, and then, the injected portion is immediately subjected to *in vivo* EP using tweezer-type electrodes and a square-pulse generator. The treated mice are kept for 7 days prior to collagenase perfusion. These resulting collagenase-dissociated hepatocytes cells are then cultured in Dulbecco's modified Eagle's medium (DMEM) supplemented with 20% fetal bovine serum (FBS), 1 *μ*g/mL of insulin, and 4 *μ*g/mL dexamethasone on a collagen-coated dish (d). One day after hepatocyte isolation, puromycin (0.3 *μ*g/mL) is added to the medium to eliminate untransfected hepatocytes and then kept for 7-10 days for generation of viable colonies.

Generally, acquiring hepatocyte cell lines from normal liver has been considered difficult, since cells tend to lose hepatocyte-specific functions soon after *in vitro* cultivation [12, 13]. Only HepaRG cells, derived from liver tumors, are known to retain hepatic functions with respect to having the ability to produce albumin and being susceptible to infection by hepatitis B virus (HBV) [14]. In earlier stages of attempts to acquire immortalized hepatocytes, many researchers employed viral infection approaches involving primary cultured hepatic cells obtained soon after isolation from liver tissue after perfusion with collagenase (Figure 1(a)). These viruses include adenovirus and SV40 virus containing oncogenic factors such as *E1A/E1B* (adenovirus) and transforming genes (*SV40*) [15–17]. Plasmid vectors containing an expression cassette for the expression of oncogenic factors (such as SV40T) have also been used for immortalization of hepatic cells [18]. In this case, electroporation- (EP-) [19] or gene delivery-related reagent-based transfection such as calcium phosphate [20–23] and liposomes [24–27] has been employed, as shown in Figure 1(a). In terms of oncogenic factors involved, DNA coding for SV40T is most frequently used. *E6* and *E7* genes from human papilloma virus (HPV) have also been employed for hepatocyte immortalization. The *E6* gene, derived from HPV16, has the ability to promote the degradation of p53 cell cycle-regulating proteins similar to SV40T [28]. On the other hand, E7 induces the degradation of the retinoblastoma protein RB, another type of cell cycle regulator [29]. Moreover, human-derived TERT (*hTERT*) gene has also been used for inducing immortalized hepatocytes [30]. In addition to the function of hTERT to maintain telomere length, it is reported to bind to transcription factors such as p65 or *β*-catenin and to regulate gene expression related to tumorigenesis [31].

At later stages of acquisition of immortalized hepatocytes, retroviral [32–44] and lentiviral [45–48] vectors are frequently used. These approaches are completely different from those reported earlier. For example, retroviral vectors can insert a single copy of a transgene into a cellular chromosome only at the periods when cells exhibit active cell division [49]. On the other hand, lentiviral vectors are active independent of cell cycle [50]. Since these vectors have high efficiency in terms of gene transfer and low cytotoxicity, they are thought to be suitable for gene transfer into cells at the interfuse stage after terminal differentiation [51]. However, construction of these vectors strictly requires cells dedicated to packaging transgenes into virus particles. Moreover, experimental equipment for containing viral particles is needed for prevention of possible contamination. These procedures are laborious and time-consuming. In contrast, gene transfer of nonviral DNA using chemical reagents or EP is much more simplified and cost-effective, although gene transfer efficiency appears to be lower than that involving viruses. Furthermore, the frequency of chromosomal integration of transgenes appears to be lower compared to viral systems. Therefore, employment of new gene delivery systems enabling effective chromosomal integration of genes of interest (GOI) into hepatocytes is required.

More importantly, many of the immortalized hepatocyte lines established by the above-mentioned technologies appear to lose hepatocyte-specific functions, as exemplified by reduced production of albumin, urea, and cytochromes, compared to the living liver. Almost all of these cells lose infectivity by HBV except the HuS-E/2 human hepatocyte cell line [52]. One reason for this failure appears to be due to *in vitro* immortalization of *in vitro* cultured hepatocytes.

## 2. Transposons as Useful Tools to Obtain Chromosomal Integration of GOI

In mammalian cells, transposon-mediated gene transposition is often performed to achieve chromosomal integration of GOI [53]. The mobility of transposons can be controlled by conditionally providing the transposase that mediates the transposition reaction. Thus, a GOI (i.e., a fluorescent marker, a small hairpin (sh)RNA expression cassette, or a therapeutic gene construct) cloned between the inverted terminal repeat sequences (called ITRs) of transposon-based vectors can be inserted into host chromosomes in a highly efficient manner.

**Figure 2:**
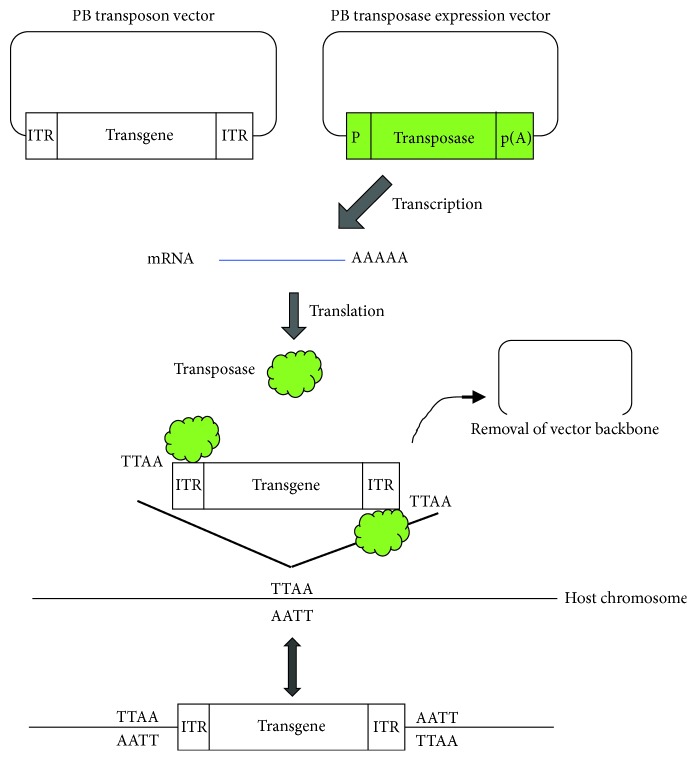
Schematic illustration of the mechanism of *piggy*Bac-based gene delivery, based on the website https://www.funakoshi.co.jp/contents/5301. Abbreviations: ITR: inverted terminal repeat; P: promoter; p(A): poly(A) sites; PB: *piggy*Bac.

*Sleeping Beauty* (*SB*) was the first transposon shown to be capable of gene transfer in vertebrate cells, and recent studies have shown that *SB* supports the full spectrum of genetic engineering techniques, including transgenesis, insertional mutagenesis, and therapeutic somatic gene transfer, both *ex vivo* and *in vivo* [54–56]. *Piggy*Bac (PB) represents an alternative transposon technique, allowing efficient integration of exogenous DNA into host chromosomes in several organisms, including humans [57–59], bovines [60], goats [61], pigs [62, 63], rats [64], mice [65], fish [66], insects [67, 68], malaria parasites [69], yeast [70], and plants [71]. This system is now widely used in gene discovery via insertional mutagenesis [72], generation of induced pluripotent stem (iPS)/embryonic stem (ES) cells [73–75], production of Tg animals [76], introduction of large transposons (>100 kb) [77], generation of stable cell lines with multiple constructs [78], and generation of genome-edited cells [79, 80]. The PB-based gene delivery system is very simple: creation of a PB transposase expression vector and transposons carrying GOI flanked by the two ITR sequences. When they are transfected into a cell, the transposase binds to the ITR to allow the GOI alone to be integrated into host chromosomal sites that contain the TTAA sequence, which is duplicated on the two flanks of the integrated fragment [81, 82]. In Figure 2, the mechanism for PB-based integration of GOI is shown schematically. Furthermore, integrated transposons can be removed by transient retransfection with the PB transposase expression vector [83, 84]. This excision is very precise, as evidenced by the typical absence of “footprint” mutations at the site of transposon excision [85].

## 3. Transposons Confer Efficient Integration of GOI *In Vivo*

As described above, previous approaches to establish immortalized hepatocytes adopted primary hepatocytes cultured as a source for gene engineering-based immortalization. In general, under the culture conditions used, isolated hepatocytes are known to show reduced viability and dramatic alterations to their gene expression profiles, probably because of drastic alterations involving cell-to-cell contact or cell-to-extracellular matrix contact [86]. This suggests that immortalization of primary cultured hepatocytes may not be the best choice for acquiring immortalized hepatocyte lines. Instead, immortalization of hepatocytes under *in vivo* condition would be the best because hepatocyte function in these *in situ* immortalized hepatocytes appears to be retained in the *in vivo* environment. We therefore considered that transfection of hepatocytes *in vivo* through liver-directed gene delivery of PB transposons may fit the above concept. As mentioned previously, the PB transposon system is useful for efficient integration of GOI into host chromosomes in cultured cells and for efficient transgenesis in mice [87]. However, little is known about whether this system is also effective *in vivo*. Recently, a number of studies have described the effectiveness of this system *in vivo*. For example, Saridey et al. [88] demonstrated that a single injection of plasmid-based PB transposons via the tail vein confers long-term (approximately 300 days after gene delivery) expression of a GOI (coding for luciferase) in the liver and lungs of mice, suggesting chromosomal integration of the GOI. Similar results were also provided by other groups who used repeated intravenous injections of PB transposons [89] or intravenous injections of hybrid PB/viral vectors [90]. We recently performed intraparenchymal injection of exogenous plasmid DNA containing a PB transposase expression vector and PB transposons and subsequent *in vivo* EP using tweezer-type electrodes to stably transfect murine pancreatic cells. This approach was originally developed to transfect pancreatic cells with naked plasmid DNA and was termed “intrapancreatic parenchymal injection for gene transfer (IPPIGT)” [91]. We found that expression of a GOI (coding for red fluorescent protein) continued for at least 1.5 months after IPPIGT (our unpublished results).

## 4. New Approaches for Generating Immortalized Hepatocyte Cell Lines Based on *In Vivo* Transfection of Hepatocyte with Transposons Carrying Immortalization Genes

**Figure 3:**
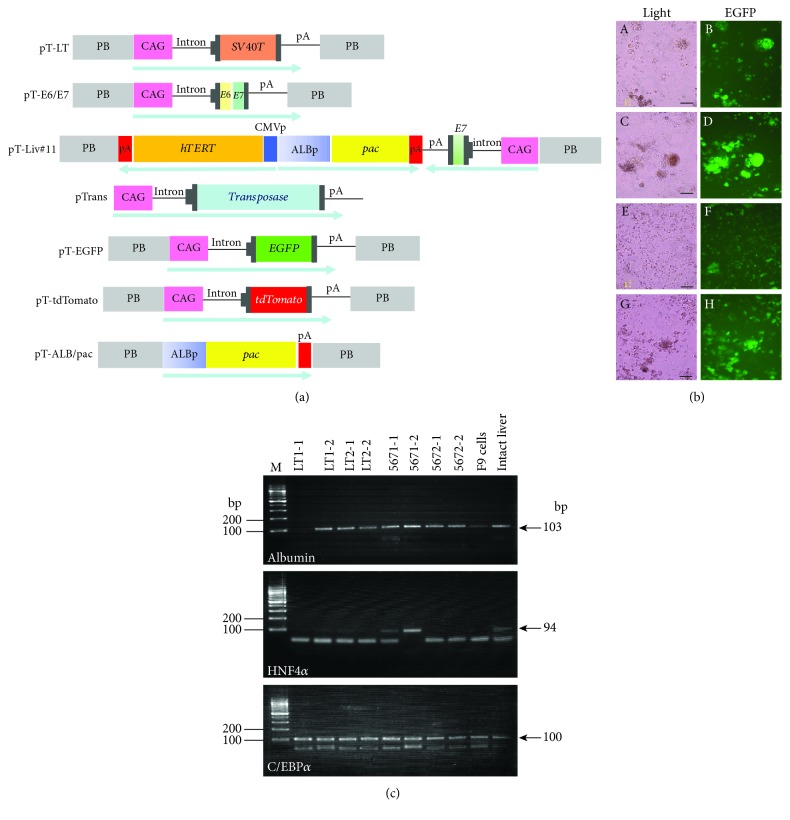
Establishing hepatocyte cell lines by *in vivo* gene transfer of PB transposons in murine liver. (a) Schematic illustrating the vectors used. pT-LT, pT-E6/E7, pT-Liv#11, pT-EGFP, pT-tdTomato, and pT-ALB/pac are transposon vectors. pTrans is a vector conferring expression of PB transposase. Upon *in vivo* gene delivery, pT-EGFP, pT-E6/E7, and pT-Liv#11 (or pT-LT and pT-ALB/pac) are cotransfected with pTrans to obtain the 567 cell line (carrying *EGFP*, *pac*, *E6*, *E7*, and *hTERT* genes) or LT line (carrying *EGFP*, *pac*, and *SV40T* genes). Arrows under each vector show the orientation of transcription in each expression unit. Abbreviation: PB: PB ITRs; CAG: chicken *β*-actin-based promoter; p(A): poly(A) signal; E6: HPV18-derived E6 protein gene; E7: HPV18-derived E7 protein gene; SV40T: SV40 T antigen gene; EGFP: enhanced green fluorescent protein gene; hTERT: hemagglutinin-tagged human telomerase reverse transcriptase gene; CMVp: cytomegalovirus promoter; ALBp: human albumin promoter; pac: puromycin acetyltransferase gene; Transposase: PB transposase; pA: poly(A) sites. (b) Cell colonies 10 days after puromycin selection. Both LT (A-D) and 567 (E-H) lines are viable, showing bright EGFP-derived fluorescence. However, there are no viable cells in the control group (data not shown). From these colonies, we obtained clonal lines called LT1-1 and LT1-2, LT2-1 and LT2-2, 5671-1 and 5671-2, and 5672-1 and 5672-2. Bar = 100 *μ*m. (c) RT-PCR analysis of hepatocyte marker gene expression in the isolated clones. The primer sets used for albumin, HNF4*α*, and C/EBP*α* were 5′-ctcaggtgtcaaccccaa-3′ and 5′-tccacacaaggcagtctc-3′, 5′-tgccaacctcaattcatcca-3′ and 5′-gctcgaggctccgtagtgtt-3′, and 5′-aagaagtcggtggacaagaacag-3′ and 5′-gttgcgttgtttggctttatctc-3′, respectively. These primer sets yielded 103 bp (for albumin), 94 bp (for HNF4*α*), and 100 bp (for C/EBP*α*) PCR products. Notably, almost all of the clones tested still exhibited expression of albumin. F9 cells: murine embryonal carcinoma cells used as negative control; intact liver: adult murine liver used as positive control. M: 100 bp ladder size marker.

**Figure 4:**
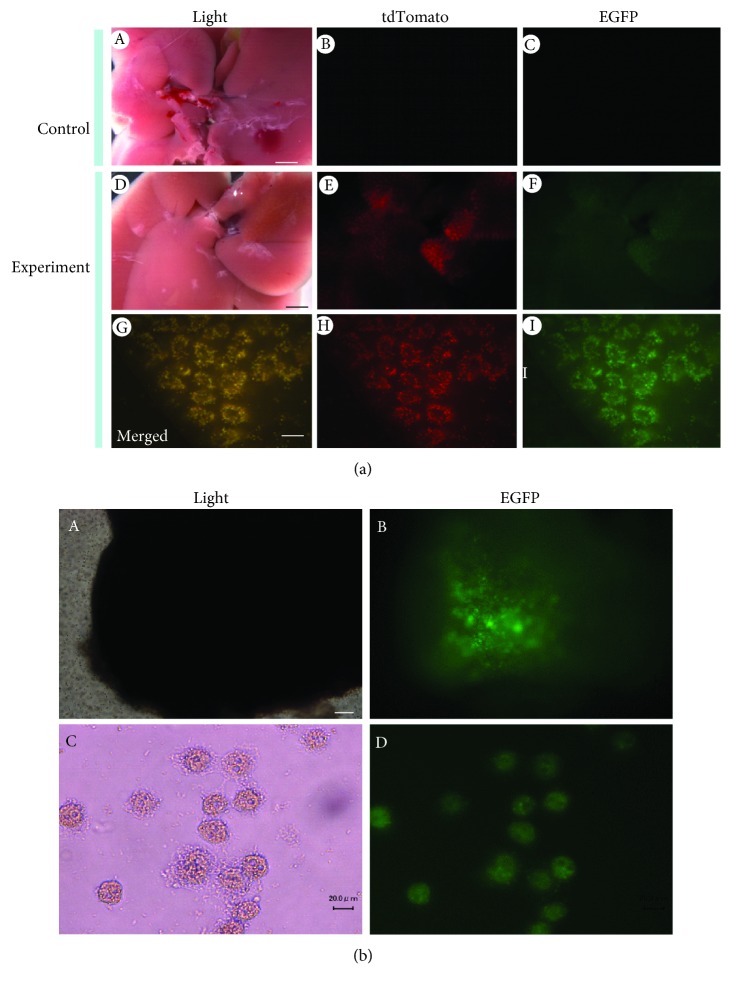
Analysis of gene expression in murine liver and hepatocytes after HGD-based gene delivery (a) and EP-based gene delivery (b). In (a), the liver is dissected 2 days after HGD with pT-EGFP, ptdTomato, and pTrans and inspected for fluorescence under a fluorescence microscope. Both green and red fluorescence are seen in the DNA-introduced experimental group (D-F) but not in the DNA-noninjected control group (A-C). Higher magnification of images in (D-F) reveals colocalization of both fluorescence colors in hepatocytes (G-I). In (b), the liver is dissected 7 days after EP with pT-EGFP, pT-Liv#11, and pTrans and perfusion with collagenase. Inspection for fluorescence in the dissected liver reveals bright green fluorescence in the electroporated region (A, B). When the fluorescent region is dissociated into single cells and subjected to culture in the presence of puromycin for 7 days, all of the surviving cells are found to exhibit green fluorescence (C, D), suggesting chromosomal integration of introduced transposons. Bar = 200 *μ*m (for a) and 20 *μ*m (for b).

Hydrodynamic (HGD) injection is a useful method for gene delivery to the liver, involving the rapid injection of a large volume of vector-containing solution into the tail vein [92, 93]. When this approach was employed for transfection with nonviral DNA in mice, the right median lobe of the liver was found to be preferentially transfected (Figure 1(b)) [94]. We recently tested whether HGD-based gene delivery using a DNA solution containing the PB transposon and a PB transposase expression construct could be used to establish prolonged GOI expression in hepatocytes of the right median lobe [94]. Coinjection of a PB transposon containing an enhanced green fluorescent protein expression unit (pT-EGFP; Figure 3(a)) and a PB transposase expression construct (pTrans; Figure 3(a)) together with the nontransposon vector, ptdTomato (conferring expression of tdTomato; [95]), resulted in EGFP expression, even after 56 days postgene delivery, while no appreciable tdTomato expression was observed in the liver sampled 28 days or more after gene delivery [95]. The result of this experiment suggests that the *in vivo* PB-based gene delivery system confers stable GOI integration in hepatocytes, indicating that HGD-based delivery of PB transposons carrying immortalizing genes may be a useful *in vivo* approach for the acquisition of immortalized hepatocyte lines. In Figure 4(a), we show an example-of-principle using HGD-based intravenous delivery of two fluorescent marker-containing transposons (pT-EGFP and ptdTomato; Figure 3(a), unpublished data). Two days after gene delivery, liver tissue was dissected for analysis of fluorescence, and two constructs were shown to have been simultaneously introduced into hepatocytes (Figure 4(a), G, H, and I). This suggests that multigene constructs can be delivered simultaneously into hepatocytes, which will be beneficial for chromosomal integration of the transposons with the aid of transposase, a product derived from the pTrans construct delivered concomitantly.

Another approach for *in vivo* immortalization of hepatocytes involves the direct introduction of foreign DNA into the liver and subsequent *in vivo* EP in combination with the use of a transposon-based gene delivery system like IPPIGT [91] (Figure 1(c)). This option is always accompanied by surgical procedures, in which the liver is exposed outside the skin. Although this procedure is often laborious, site-specific gene delivery is possible and easy because researchers can control this under observation using a dissecting microscope. Direct introduction of transposon-based vectors carrying immortalizing genes (i.e., *SV40 T* and *hTERT*) into animal livers would result in the *in vivo* establishment of immortalized hepatocyte cell lines. In Figure 4(b), we show an example-of-principle (unpublished data) for the possible isolation of immortalized hepatocyte cell lines using this novel approach. First, a small volume (2–3 *μ*L) of solution containing two PB transposons, pT-EGFP and pT-Liv#11, as well as pTrans, each at a concentration of 100 ng/*μ*L, is introduced into the internal area of the murine liver by inserting a glass micropipette under observation using a dissecting microscope and subsequently injecting the solution using the procedure described by Sato et al. [91] (Figure 1(c)). A small amount of India ink is added to the solution to visualize the location of injection sites. pT-Liv#11 is a transposon vector carrying *hTERT*- and HVP18-derived *E7* expression units, together with a puromycin acetyltransferase gene (*pac*) expression unit (under the control of an albumin promoter construct) (Figure 3(a)). Simultaneous delivery of these three vectors into a cell should result in EGFP-derived green fluorescence and resistance against puromycin in cells of the hepatocyte lineage. For transfection with pT-E6E7 or pT-LT (Figure 3(a)) together with pTrans and pT-ALB/pac (Figure 3(a)), a plasmid carrying the *pac* gene under the control of the albumin promoter is cotransfected in the liver. The injection site is then subjected to *in vivo* EP using tweezer-type electrodes (Figure 1(c)). Eight square electric pulses of 50 V with a constant time of 50 milliseconds (ms) are applied using a pulse generator. With this treatment, some hepatocytes receiving the foreign DNA may be stably transfected. Seven days after gene delivery, the liver is dissected after perfusion with Hanks' balanced salt solution (HBSS) without Ca^2+^ or Mg^2+^ but containing 1 mg/mL collagenase (Figure 1(c)). The injected portion is easily recognizable by the expression of a fluorescent marker visible under a fluorescent dissecting microscope. In Figure 4(b), A and B, bright fluorescence is easily discernible in the electroporated area. The dissected liver can be dissociated into single cells by teasing apart in HBSS containing collagenase, followed by further incubation at 37°C for more than 1 h to further dissociate the cells prior to culturing in hepatocyte culture medium containing hepatocyte growth factor (HGF) and dexamethasone. To obtain immortalized hepatocytes, the dissociated cells obtained by collagenase perfusion are seeded (5 × 10^6^) into a 6 cm collagen-coated cell culture dish with hepatocyte culture medium. Puromycin is then added to the medium at a concentration of 2 *μ*g/mL, and cells are cultured for 7 days to obtain recombinant cells. The emerging surviving cells (colonies) are picked and seeded onto a collagen-coated 24-well plate and cultured until subconfluency and are then propagated by seeding 3–5 × 10^5^ cells onto a 35 mm collagen-coated dish. The medium is changed every 2 days until subconfluency. The surviving cells should contain transposons in their genomes, conferring resistance against the selective drug and driving cell proliferation due to the expression of the exogenous immortalizing genes. Fluorescence in the surviving hepatocytes (7 days after puromycin treatment) is shown in Figure 4(b), C and D. Notably, almost all the cells are fluorescent, suggesting stable transfection with both the pT-EGFP and pT-Liv#11 transposons. For further propagation, these engineered hepatocytes must be cultured in medium containing factors (i.e., insulin and dexamethasone) that support hepatocyte growth (Figure 1(d)). Several hepatocyte lines (called LT1-1 to LT1-2, LT2-1 to LT2-2, 5671-1 to 5671-2, and 5672-1 to 5672-2) were eventually obtained, all of which survived after puromycin treatment and exhibited EGFP fluorescence (Figure 3(b)). LT1-1 to LT1-2 (Figure 3(b), A, B, C, and D) and LT2-1 to LT2-2 were derived from liver tissue transfected with pT-EGFP, pT-LT, pT-ALB/pac, and pTrans. 5671-1 to 5671-2 (Figure 3(b), E, F, G, and H) and 5672-1 to 5672-2 were derived from liver cells transfected with pT-EGFP, pT-Liv#11, pT-E6E7, and pTrans. Analysis of these established lines by RT-PCR demonstrated that almost all the lines expressed hepatocyte marker genes, such as albumin (Figure 3(c)). Since albumin expression is an important marker of hepatocyte function, the resulting lines are considered to retain the properties of functional hepatocytes. Our future efforts to characterize these established lines would involve examination of the expression of other hepatocyte-specific proteins and urea synthesis. Performing RNA sequencing (RNA-Seq) analysis would also be useful to examine whether our lines indeed resemble hepatocytes at the transcriptional level *in vivo*.

## 5. Perspective for Translational Medicine

There is an increasing demand for human hepatocytes differentiated from pluripotent stem cells (as exemplified by ES/iPS cells) for translational medicine [96]. Since the first report by D'Amour et al. [97], who demonstrated the ability of activin A to induce efficient differentiation of human ES cells to definitive endoderm, extensive studies have been carried out on the induction of ES/iPS cell differentiation towards an endodermal lineage. For example, generation of hepatocyte precursors from endodermal cells is achieved by combined treatment with fibroblast growth factor 4 (FGF4) and bone morphogenetic protein 2 (BMP2) [98] or FGF1/2/4 and BMP2/4 [99]. Differentiation of hepatocyte precursors into functional hepatocyte-like cells (HLCs) has typically been achieved by treatment with factors such as HGF, oncostatin M, and dexamethasone [100, 101]. The resulting HLCs have the potential to cure a patient with liver failure through hepatocyte transplantation. Nagamoto et al. [102] demonstrated that transplantation of human iPS cell-derived HLC sheets (created by culturing iPS cells in a temperature-responsive culture dish) into the liver of model mice that show acute liver failure resulted in increased survival rates. However, the use of iPS cells for therapeutic purposes still retains immunogenic and tumorigenic potential [103], and several groups have tried to apply immortalized human hepatocytes for clinical use to bypass the concerns related to the nature of iPS cells. However, ethical issues still remain. For example, there is concern regarding potential tumor generation after transplantation of immortalized hepatocytes, although subcutaneous injection of immortalized hepatocytes into severe combined immunodeficiency (SCID) model mice did not induce tumor development [104]. However, despite the report, the potential for tumorigenesis cannot be completely excluded since the genomes of the immortalized hepatocytes still retain immortalizing genes. Urschitz and Moisyadi [105] suggested that these genes chromosomally integrated through PB-mediated gene delivery could be completely removed before cell transplantation by transient retransfection with a transposase expression vector. Totsugawa et al. [106] used tamoxifen for the Cre/*loxP*-mediated removal of a floxed immortalizing gene from immortalized hepatocytes after transfection with a gene coding for tamoxifen-dependent Cre recombinase.

Notably, the present technology appears to be confined to smaller experimental animals such as mice and rats (shown in Figures 3 and 4). However, we think that it is also theoretically applicable to larger animals (such as the pig and cow), as well as humans, particularly since the development of *in vivo* liver-targeting gene delivery methods for gene therapy. Interestingly, some reports have described HGD-based gene delivery in the pig [107, 108]. These experiments were aimed at developing techniques related to gene therapy as basic research but hold potential for the acquisition of functional immortalized porcine hepatocytes. In this context, pigs may be a useful resource to examine whether our present strategy will work well. Indeed, we successfully obtained immortalized hepatocytes from dissected porcine liver using our vector system (shown in Figure 3(a)), although the efficiency was very low (data not shown).

## 6. Conclusion

To date, an enormous number of immortalized cell lines have been generated. Most of these are derived from transfection of primary cells with vectors carrying genes for immortalizing factors or by primary culture of tissues/organs dissected from Tg mice carrying immortalizing genes. Our present idea, based on site-directed introduction of chromosomal-integrating transposons into living animals, appears to be unique and simple and will provide an additional, easy way to establish novel, immortalized cell lines (including immortalized hepatocytes), which are often refractory to *in vitro* transfection with vectors carrying immortalizing genes.
